# The Complex Genetic and Epigenetic Regulation of the Nrf2 Pathways: A Review

**DOI:** 10.3390/antiox12020366

**Published:** 2023-02-03

**Authors:** Joe M. McCord, Bifeng Gao, Brooks M. Hybertson

**Affiliations:** 1 Pathways Bioscience, Aurora, CO 80045, USA; 2 Department of Medicine, Division of Pulmonary Sciences and Critical Care Medicine, University of Colorado Anschutz Medical Campus, Aurora, CO 80045, USA

**Keywords:** Nrf2, epigenetic regulation, pathway analysis

## Abstract

Nrf2 is a major transcription factor that significantly regulates—directly or indirectly—more than 2000 genes. While many of these genes are involved in maintaining redox balance, others are involved in maintaining balance among metabolic pathways that are seemingly unrelated to oxidative stress. In the past 25 years, the number of factors involved in the activation, nuclear translocation, and deactivation of Nrf2 has continued to expand. The purpose of this review is to provide an overview of the remarkable complexity of the tortuous sequence of stop-and-go signals that not only regulate expression or repression, but may also modify transcriptional intensity as well as the specificity of promoter recognition, allowing fluidity of its gene expression profile depending on the various structural modifications the transcription factor encounters on its journey to the DNA. At present, more than 45 control points have been identified, many of which represent sites of action of the so-called Nrf2 activators. The complexity of the pathway and the synergistic interplay among combinations of control points help to explain the potential advantages seen with phytochemical compositions that simultaneously target multiple control points, compared to the traditional pharmaceutical paradigm of “one-drug, one-target”.

## 1. Introduction

**Figure 1:**
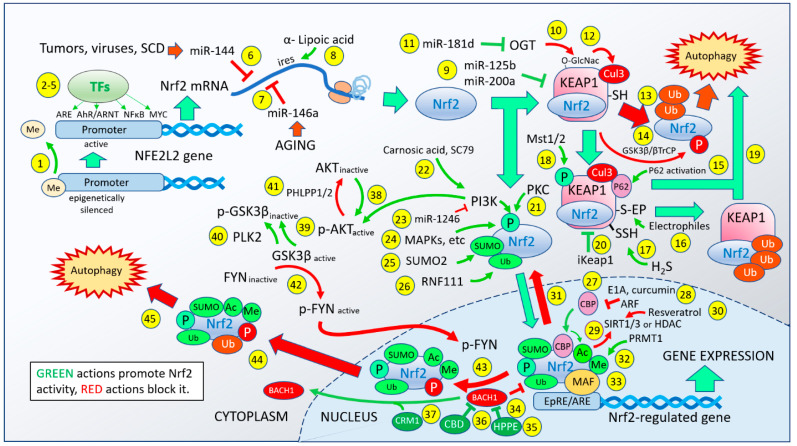
This figure displays the sequence of events in the journey of an Nrf2 molecule from the transcription of the *NFE2L2* gene through its translation, its encounter with and destruction by Keap1 (or, alternatively, its avoidance of Keap1), its decoration by a number of possible covalent modifications, its entry into the nucleus, its further decoration and association with cofactors, its promotion of EpRE/ARE-regulated genes and, finally, the mechanisms in place to terminate its role as a transcription factor, to expel it from the nucleus and send it to the proteasome for destruction. Forty-five discreet actions are numbered along the way and discussed in the text, with actions supporting Nrf2 activity shown in green, while those that decrease Nrf2 activity are shown in red.

In the past two decades, the sequence of events leading to the activation and expression of genes controlled by the actions of the Nrf2 transcription factor has steadily expanded. Figure 1 is an attempt to represent the multitude of biological events now known to modify the ultimate ability of Nrf2 to bind to and control the expression of genes regulated by the EpRE and ARE promoter sequences [1]. The figure is not intended to be exhaustive or final. Rather, its intent is to illustrate the complexity of the process and the potential ways in which the Nrf2 response can be tweaked, modified, amplified, or repressed, including multiple actions that affect the duration of its actions and the mechanisms of termination of those actions. Together, the sheer complexity of the process reflects the evolutionary importance of the pathway, along with the considerable amount of “evolutionary attention” it has received to achieve its current state. The 45 numbered points of intervention include actions that increase the ability of Nrf2 to do its job (shown by green indicators) and actions that interfere with it, attenuate it, or shut it down (shown by red indicators). Interestingly, the positive vs. negative events are interspersed at nearly all stages of the process, suggesting that organism-wide regulation of Nrf2 probably proved to be inadequate early on, prompting local control in the various organs and tissues. The number of genes that have evolved to be under the control of Nrf2 is surprisingly large. We have previously reported that in human umbilical vein endothelial cells more than 3000 genes show statistically significant regulation by Nrf2 activation, with a 1.5-fold difference in expression from untreated cells [2]. The potential range of physiological effects that might result from a genetic perturbation of this magnitude is difficult to fathom, explaining the huge array of conditions related to health, disease, aging, and stress survival that have been ascribed to changes in Nrf2 activity. The most commonly used description for these genes is that they are “stress-related”. The types of stress include oxidative stress, metabolic stress, age-related stress, mechanical stress, and even psychological stress. No wonder, then, that the control of Nrf2 has evolved to be locally determined—the genes responding to a sunburn are expected to be quite different from those responding to major depressive disorder, to a coronary artery infarction, or to a viral infection. The activation of Nrf2 is not “one size fits all”. Beyond that, many scientific reports have treated Nrf2 activation as a binary event, completely ignoring the degree to which Nrf2 is activated and the types and numbers of post-translational modifications that characterize the Nrf2 that eventually makes its way to the nuclear DNA. As more and more pharmaceutical interest is directed toward Nrf2 activators, we believe the details of exactly how, where, and when the activation takes place must become central to the discussion.

While the rapidly growing interest in Nrf2 is driven by the physiological effects resulting from the induction or repression of the genes it controls, those downstream effects are outside the scope of this review, which focuses on the events that affect the ability of the Nrf2 molecule per se to be expressed, modified, processed, activated, and disposed of by various cellular mechanisms.

## 2. The Nrf2 Activation Pathway

The sequence of events shown in Figure 1 follows the Nrf2 molecule from its creation to its demise, including 45 known events that may affect its ultimate ability to regulate gene expression. These events include transcription, translation, translocation, sequestration and release, post-translational modifications, binding, partnering, promotion or repression, and eventual expulsion from the nucleus to be degraded. Nrf2 is encoded by the *NFE2L2* gene. A recent review [3] describes the *NFE2L2* promoter as containing six CpG methylation sites, indicated as Point 1 in Figure 1. Epigenetic regulation of Nrf2 expression by hypermethylation at these sites has been seen in primary epithelial cells from patients with chronic obstructive pulmonary disease (COPD) who showed decreased Nrf2, increased reactive oxygen species, lipid peroxidation, and ferroptosis [4], demonstrating a role for this point of regulation in clinical medicine. Furthermore, resveratrol has been shown to reverse methylation of the *NFE2L2* promoter caused by high glucose in HepG2 cells, suggesting a therapeutic approach to nonalcoholic fatty acid liver disease (NAFLD) at this control point [5]. The *NFE2L2* promoter also contains transcription factor binding sites (Points 2–5) that include the antioxidant response element (ARE), which allows Nrf2 the ability to regulate its own transcription, as well as binding sites for the aryl hydrocarbon receptor/aryl hydrocarbon receptor nuclear translocator (AhR/ARNT), for NF-κB, and for MYC [3]. The presence of these additional binding sites permits some overlap of gene expression profiles among these transcription factors, as discussed later. Once the Nrf2 mRNA has been transcribed, translation may proceed. However, translation may be impeded by HIV-1 transgene expression in vivo in rat alveolar epithelial cells [6], as indicated at Point 6. The suppression is mediated by miR-144—a microRNA—and could be reversed by antagonizing miR-144 or by activating Nrf2 with PB123, suggesting that intervention at this control point might decrease lung-specific morbidity and mortality in persons living with HIV [6]. Li et al. [7] have shown that miR-144-mediated Nrf2 silencing inhibits fetal hemoglobin expression in those with sickle-cell disease. High HbF production can prevent a sickle crisis from occurring. Individuals producing the lowest levels of HbF showed eightfold upregulation of miR-144, suggesting that an miR-144 antagomir or activation of Nrf2 might be clinically useful in the management of sickle-cell disease. Similar relationships between miR-144 and Nrf2 have been noted in the context of obesity-associated insulin resistance [8] and oxygen–glucose deprivation/reoxygenation-induced neuronal injury [9]. Another microRNA, miR-146a, may play a major role in the age-related decline in Nrf2 expression at Point 7. Smith et al. [10] found that in cultured hepatocytes from young and old rats there were no changes in Nrf2 mRNA levels but there was a significant decline in Nrf2 protein and in Nrf2-dependent gene expression in cells from the old animals, implying that something was interfering with the translation. The inhibiting entity was identified as miR-146a. A substantial and rapidly growing body of literature exists on the roles of miRNAs in the regulation of Nrf2, and these roles are admittedly underrepresented in Figure 1. A number of reviews have surveyed the roles of miRNAs in the regulation of the Nrf2 pathway [11] and their specific roles in Alzheimer’s disease [12], heart failure [13], and ischemia/reperfusion injury [14]. Another important control point in the pathway that occurs at the Nrf2 mRNA stage is the existence of an internal ribosome entry sequence (IRES) in the 5’ untranslated region of the human Nrf2 mRNA, first noted by the Kong laboratory [15]. The IRES allows the translational machinery to bypass the cap at the 5’ end of the messenger RNA, which normally halts translation in a cell under high stress conditions. The Hagen laboratory found that alpha-lipoic acid (Point 8) could cause rapid and substantial translation of Nrf2 by activating this IRES to provide additional Nrf2 via new synthesis in response to abrupt-onset oxidative stress [16]. This mechanism requires neither increased transcription of the *NFE2L2* gene nor avoidance of Keap1-dependent depletion of Nrf2 in the cytosol. At this point, we begin to observe the complexities and redundancies in this exquisitely regulated pathway—if the normal supply chain is compromised, an emergency alternative or workaround may come into play. Once the Nrf2 protein enters the cytosol, it may be bound by the Keap1 molecule. Several controls may be exerted at the level of the Nrf2–Keap1 interaction. MiR-125b (Point 9) has been found to protect retinal pigment epithelial cells from oxidative damage by substantially inhibiting Keap1 expression and enhancing Nrf2 expression [17]. Similarly, miR-200a has been found to inhibit Keap1 expression, and it also improves diabetic endothelial dysfunction [18]. These actions increase Nrf2’s nuclear translocation, as the Nrf2 molecules outnumber the Keap1 molecules, allowing Nrf2 to proceed to the nucleus unimpeded. Furthermore, increased expression of miR-125b significantly increased superoxide dismutase expression and reduced the formation of reactive oxygen species and malondialdehyde reflecting Nrf2 activation, suggesting that this action ultimately resulted in increased expression of Nrf2-regulated genes. At Point 10 in the pathway, a post-translational modification of Keap1 at serine 104 by O-linked N-acetylglucosamine (GlcNAc) transferase (OGT) [19] activates efficient ubiquitination of its bound Nrf2 by the Keap1–Cul3 E3 complex [20] (Point 12), sending the Nrf2 away for proteasomal degradation. This mechanism has been implicated in high-phosphate-induced vascular calcification leading to chronic kidney disease [21]. This destructive act by OGT against Nrf2 may be blocked (Point 11) by the action of miR-181d—an inhibitor of OGT [22] found to target the 3’UTR of OGT mRNA to prevent OGT expression in ovarian cancer, elevating Nrf2 activation and providing cisplatin resistance. Thus, this single seemingly obscure control point in the Nrf2 activation pathway appears sufficient to decrease Nrf2 activity leading to kidney disease, or to elevate Nrf2 activation in ovarian cancer cells sufficiently to cause resistance to an anticancer drug. This is a remarkable illustration of the delicacy of the balance and the number of pathogenic ways in which that balance may be upset. The ability of the Keap1–Cul3 E3 complex to tag Nrf2 for degradation by the proteasome (Point 13) appears to be just as important to the healthy survival of cells as is the ability of Nrf2 to escape from proteasomal degradation and to translocate to the nucleus. Either action is apt to cause pathogenicity when it malfunctions. Point 14 represents another layer of regulation to enhance the proteasomal degradation of Nrf2. GSK-3β can phosphorylate a group of serine residues in the Neh6 domain of Nrf2, stimulating its association with SCF/β-TrCP, promoting proteasomal degradation and shortening its half-life in the cell [23,24]. Recently, a small molecule (PHAR) has been described that selectively inhibits the interactions between GSK-3β, β-TrCP, and phosphorylation sites on Nrf2 that would tag it for degradation [25]. Accordingly, PHAR allows Nrf2 to avoid degradation, allowing nuclear translocation to occur. This is not the only point at which GSK-3β promotes the destruction of Nrf2; we can also see it make another important appearance much later in the pathway. The activation of p62 (encoded by *SQSTM1*) enables a p62/Keap1/Nrf2 signaling pathway (Point 15). A number of factors seem capable of activating autophagy via p62, including resveratrol [26,27], lycopene [28], and hydrogen sulfide [29]. The activation of p62 appears to take place via AMPK phosphorylation at serine-351 in the mouse protein, which then binds to Keap1 and escorts it to the proteasome for degradation, allowing Nrf2 to avoid degradation by Keap1 [30]. This mechanism increases Nrf2 activation and has been shown to protect liver cells from saturated-fatty-acid-induced apoptotic cell death [30]. Interestingly, Nrf2 strongly upregulates *SQSTM1*, so this increased availability of p62 may be an additional mechanism whereby Nrf2 can increase its own availability.

**Figure 2:**
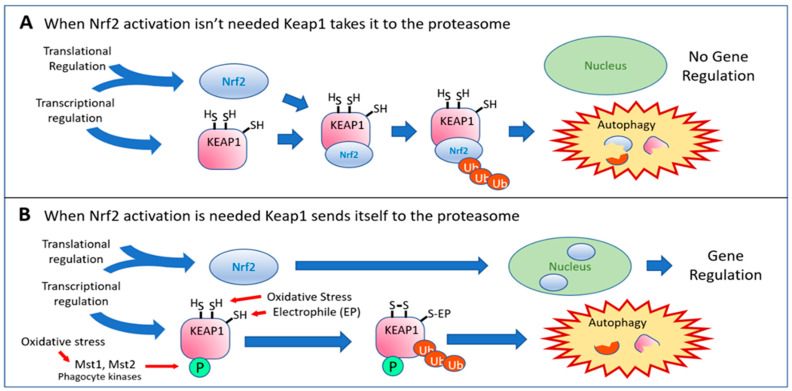
(**A**) When Keap1 is present, newly synthesized Nrf2 is immediately bound, ubiquitinated, and the complex is degraded by the proteasome. (**B**) When oxidative stress or an electrophilic Nrf2 activator is present, the structural changes enable Keap1 to ubiquitinate itself. It is then eliminated by the proteasome, and the newly synthesized Nrf2 has an unimpeded path to the nucleus. The macrophage kinases Mst1 and Mst2, activated by oxidative stress, tag Keap1 for destruction to increase Nrf2’s production of antioxidant enzymes to allow phagocytes to better protect themselves when activated.

By far the most studied mechanism to prevent Keap1 from degrading Nrf2 has been the electrophilic modification of its cysteine residues (Point 16). Literally hundreds of papers have described electrophilic small molecules (known as Michael acceptor molecules) that react avidly with several cysteines of the Keap1 protein, resulting in regulation of the Nrf2 and/or NF-κB pathways. Most of these molecules are naturally occurring phytochemicals or synthetic molecules based on these naturally occurring compounds. Recently, Liang et al. compared 50 such natural compounds [31], and several pharmaceutical Nrf2 activators are based on the structures of these plant-produced compounds [32,33,34,35,36,37]. Hydrogen sulfide—a natural metabolite produced by cystathionine gamma-lyase (which is strongly induced by Nrf2 activation)—can also modify cysteine 151 in Keap1 by S-sulfhydration [38], protecting cells from oxidative stress and premature senescence at Point 17 (in addition to its aforementioned ability to activate p62 at Point 15 [29]). The phosphorylation of Keap1 by macrophage kinases Mst1 and Mst2 (Point 18) represents a potentially important regulatory control point that may serve as an alternative sensor to sulfhydryl modification of Keap1. The Mst1/2 kinases are not specific to cells of the immune system but are found in many tissues [39]. They are themselves activated by oxidative stress, perhaps responding to different levels of oxidative stress or to different types of oxidants, illustrating how the pathway has evolved its intricate capacity for regulation to accommodate the needs of specific cell types, tissues, or organs. Wang et al. [40] speculated that macrophages—and perhaps other immune cell types that generate potentially damaging levels of reactive oxygen species as they go about their business of protecting us from invading pathogens—may need a special boost of antioxidant enzymes to protect themselves from their own artillery. The macrophages possess kinases (Mst1 and Mst2) that are activated by the oxidative environment and proceed to phosphorylate their target, Keap1, blocking it from ubiquitinating and degrading Nrf2, such that Nrf2 can accumulate, translocate to the nucleus, and protect the macrophages. Because Nrf2’s availability declines with age, the authors further posit that the decline of this mechanism may account for the age-related decline in immune function. This mechanism is shown in Figure 2.

From the 1999 landmark discovery of the ability of Keap1 to repress the activity of Nrf2 by Itoh et al. [41] until a paper published in 2005 by Eggler et al. [42], the most widely accepted (and still used) model for how Nrf2 ends up in the nucleus after electrophilic modification of Keap1 cysteines was the belief that the covalent modification of Keap1 diminished its affinity for Nrf2, and that the released Nrf2 was then free to diffuse into the nucleus. Eggler et al. [42] showed that release of bound Nrf2 probably never actually occurs. What happens upon reaction with Keap1 sulfhydryl groups is that Keap1 ceases to ubiquitinate Nrf2 and instead ubiquitinates itself, sending itself (and the bound Nrf2) to the proteasome for degradation (Point 19). The molecular changes involved in this transformation have been reviewed by Turpaev [43]. As the Keap1 concentration rapidly diminishes, newly synthesized Nrf2 finds no Keap1 to bind it, allowing it free access to the nucleus [42]. These events are illustrated in Figure 2. Importantly, new synthesis of Nrf2 can be achieved rapidly by activating IRES-mediated translation of pre-existing mRNA, as discussed above for Point 8. Regulation at the transcriptional level is not required. This correction to our understanding of the mechanism is an important clarification but, because of the rapid flux of Nrf2 via synthesis and degradation, it does not really affect the subsequent events. The Nrf2 entering the nucleus is not previously bound Nrf2 but, rather, newly synthesized Nrf2 that has never been bound by Keap1. The rapid rate of synthesis of Nrf2 and the equally rapid ubiquitination and destruction of Nrf2 by Keap1 [44] render the practical differences between the two mechanisms minimal. Because most small-molecule activators of Nrf2 act via the mechanism depicted in Figure 2, there is appropriately some concern regarding the fact that poorly targeted, non-targeted, or overly exuberant electrophilic reactivity can deplete glutathione and modify essential thiol groups on numerous important molecules within the cell, producing unwanted adverse effects. Curcumin [45] and sulforaphane [46,47]—both widely used “antioxidant” supplements—and dimethyl fumarate [48]—the pharmaceutical therapy marketed as Tecfidera^®^ for the treatment of multiple sclerosis—all show this ability to deplete glutathione. Curcumin (from turmeric) has been implicated in causing severe liver injury, especially when combined with piperine, which is increasingly used in dietary supplements to dramatically increase the absorption of curcumin [49,50]. CDDO, CDDO-Me, and CDDO-Im, also under development as pharmaceutical Nrf2 activators, have all been shown to deplete mitochondrial glutathione rapidly and selectively, triggering apoptosis [51]. Carnosic acid may represent an important exception among electrophiles capable of activating Nrf2. Takumi Satoh, Stuart Lipton, and coworkers found that carnosic acid acts as a prodrug [52,53,54], possessing only very weak electrophilic properties until it is oxidized to its quinone form under conditions of increased production of reactive oxygen species, as would exist in an injured or inflamed tissue. Only then does it act as an electrophile at Point 16. We have found that this desirable property of carnosic acid dominates the behavior of PB125—a composition comprised of three synergistic Nrf2 activators that shows little evidence of electrophilic toxicity [55]. Interest is rapidly increasing in a distinctly different class of Nrf2 activators that are based on interfering with the protein–protein interaction between Nrf2 and Keap1, without any covalent modifications to Keap1, as indicated at Point 20. These are discussed later under Future Directions.

Returning to Figure 1, we can see that Points 9–20 are focused entirely on enhancing or inhibiting Keap1’s ability to block all actions of Nrf2 by sending it to the proteasome. Assuming that prevailing conditions require Nrf2 activation and that Keap1’s efforts are successfully thwarted, Nrf2 now has an opportunity to enter the nucleus, only to encounter additional positive and negative control points. The phosphorylation of serine-40 in Nrf2 by protein kinase C (Point 21) has been reported to weaken the binding of Nrf2 to Keap1, promoting nuclear translocation [56]. Carnosic acid [57,58] and the small molecule SC79 [59,60] activate PI3K (Point 22), which can phosphorylate Nrf2, promoting nuclear entry; perhaps more importantly, they can also phosphorylate and activate AKT—an action that we can see later in the pathway for determining how long Nrf2 may remain in the nucleus. The activation of PI3K can be blocked by miR-1246 [61] (Point 23). Furthermore, several additional kinases—including MAPKs, CK2, PERK, and CDK5 (Point 24)—can promote Nrf2 activation via phosphorylation of multiple threonine and serine residues, as comprehensively reviewed by Liu et al. [62]. It is not clear in every case whether they act by directly phosphorylating Nrf2, or perhaps by phosphorylating CBP (Point 27) and enhancing its positive effect on Nrf2 transcription [63]. Two SUMOylation sites—lysine-111 and lysine-533—have been identified in human Nrf2 for covalent attachment of SUMO-2 (Point 25)—which enhances the half-life of the protein—its nuclear translocation, and for efficient transcriptional activity [64]. Finally, at Point 26, Nrf2 is ubiquitinated at Lys-48 by Arkadia/RNF111, which results in the stabilization of Nrf2, contrary to the usual action of ubiquitination, which targets the tagged protein for proteasomal degradation [65]. After entering the nucleus, additional factors come into play regulating the binding, efficiency of transcription, and potentially the ability to discriminate among sequence variants of the vast number of EpRE/ARE promoter sequences available in the genome. Two domains in the Nrf2 structure—Neh4 and Neh5—can bind to CREB-binding protein (CBP) at Point 27 [66]. CBP is encoded by *CREBBP* in humans and is frequently referred to as p300/CBP because p300, which is encoded by *EP300* in humans, shares a high degree of homology and overlapping enzymatic activities with CBP [67]. One or both sites on Nrf2 may be occupied, and substantial synergy can be observed with simultaneous binding. Sodium butyrate has been reported to enhance the binding of p300 to the promoter of the *NFE2L2* gene, but it is not clear whether it enhances p300 to other EpRE/ARE promoters [68]. Interestingly, p300/CBP is inhibited by an adenovirus protein, E1A [66]. Thus, blocking the positive control of Nrf2 at Point 27 by CBP inhibition may be a convenient way for viruses to interrupt the antiviral responses controlled by Nrf2 [55]. CBP activity is also required for neuronal resistance against ischemic injury [69], suggesting that its ability to enhance Nrf2’s transcriptional efficiency might be the mechanism for the neuroprotection. It is also interesting to note that curcumin—the active ingredient of turmeric, often used as an antioxidant supplement—is an inhibitor of CBP [70] like the viral E1A protein at Point 28. This may partially explain the checkered reputation of curcumin and its clear and potent toxicity at high dosages [45,50], as discussed above in view of its strong electrophilicity. Sun et al. [67] found that p300/CBP not only binds to Nrf2 but also catalyzes the acetylation of multiple lysine residues in the protein through its histone acetyltransferase (HAT) activity (Point 29). The increased transcriptional activity appears to reflect these covalent modifications rather than the binding of CBP to Nrf2 per se. Acetylation of transcription factors by HATs and deacetylation by HDACs are common reversible post-translational modifications with important roles in the regulation of signal transduction pathways [71,72]. Furthermore, ARF—an alternative reading frame product of the *CDKN2A* gene—has been proposed as a checkpoint for oxidative stress responses by inhibiting CBP-dependent acetylation of Nrf2 at Point 28 [73]. At Point 29, we can see the reversibility of Nrf2 acetylation catalyzed by SIRT1 or HDAC—which have deacetylation activity—and by resveratrol (Point 30), which is an activator of SIRT1, as reported by Kawai et al. [74]. In keeping with this observation, resveratrol was observed to downregulate Nrf2 expression by 70% in young lung fibroblast cells, and a similar response of >50% downregulation was observed for quercetin (another frequently used “antioxidant” supplement) [75]. Using multiple techniques, Kawai et al. showed that acetylated Nrf2 favors nuclear location, and prevention or reversal of acetylation diminishes binding to the promotor and favors return to a cytosolic location (Point 31) [74]. It is noteworthy that Ding et al. [76] have reported exactly the opposite effect—that SIRT1 promotes deacetylation, which activates Nrf2 activity. Similarly, Mercado et al. [77] concluded that decreased histone deacetylase 2 (HDAC2) impairs Nrf2 activation by oxidative stress but, given the complexity of the pathway and the obvious role played by acetylation/deacetylation of actual histones in regulating transcription of *NFE2L2* versus acetylation of the Nrf2 protein, the discrepancy may be due to comparing “apples-to-oranges”, and more specific experimental designs may be necessary to reconcile the conflicting observations. Another covalent modification of Nrf2—methylation of Arg-437—is catalyzed by protein arginine methyltransferase-1 (PRMT1) at Point 32 [78]. This modification moderately increases DNA binding and transactivation. The elimination of the methylation site caused an approximately 34% decrease in transcriptional efficiency using the ARE sequence taken from the *HMOX1* gene promoter. The study presented evidence that methylation of Nrf2 by PRMT1 is required for the recruitment of p300/CBP at the *HMOX1* ARE promoter, noting that among the four signature Nrf2-regulated genes examined, certain genes (i.e., *HMOX1* and *GCLM*) were affected more than others (i.e., *NQO1* and *TXNRD1*) by the absence of methylation [78]. This is one of the few studies to date suggesting that post-transcriptional modification of Nrf2 may be able to significantly change the transcription factor’s gene expression profile.

To bind efficiently to the EpRE/ARE elements and activate gene transcription, Nrf2 must form a heterodimer with a small Maf protein, and MafF, MafG, and MafK seem to be largely interchangeable for this role at Point 33 [79]. In the absence of Nrf2, the small Maf protein may form a homodimer, which can also bind to many ARE sites and function as a repressor. The Nrf2–MafG heterodimer displays unusually tight binding to the ARE, but Sengoku et al. [80] suggested that at high concentrations of Nrf2 (as may be present in some cancers with Keap1 mutations) Nrf2 may be able to activate transcription without the assistance of its Maf partner. Bach1 is a transcription factor of the same family as Nrf2—the Cap’n’collar (CNC) basic leucine zipper (b-Zip) family [81]—and it can also form heterodimers with small Maf cofactors and can recognize and bind to ARE elements. Bach1, however, nearly always functions as a repressor rather than an activator (Point 34), leading Ahuja et al. [82] to propose that small-molecule antagonists of Bach1—especially a substituted benzimidazole designated HPPE (Point 35)—might be attractive candidates for the upregulation of ARE-driven genes, essentially activating Nrf2 in a way that avoids the electrophilic toxicity shown by many of the traditional Keap1 inhibitors [83]. Similarly, cannabidiol (CBD)—a non-psychotropic phytocannabinoid with therapeutic potential—has been found to upregulate many Nrf2-regulated genes in microglial cells [84]. In keratinocytes, the mechanism of the weak induction of antioxidant pathways has been shown to be via inhibiting Bach1 (Point 36) [85]. Bach1’s suppression of ARE elements can also be alleviated by exporting it from the nucleus [86]—an action brought about by nuclear export protein Crm1 (Point 37), also known as exportin1, which is encoded by the *XPO1* gene in humans.

The primary mechanism for determining the duration of Nrf2’s presence in the nucleus is regulated from outside the nucleus and involves the activity of PI3K, which is activated by many phytochemical Nrf2 activators, such as carnosic acid at Point 22 [57]. Salazar et al. [87] determined the link between the PI3K/AKT pathway and increased Nrf2 activity resulting from increased duration of Nrf2 in the nucleus, and that link was GSK-3β. PI3K phosphorylates and activates AKT at Point 38, which phosphorylates GSK-3β at Ser-9, inactivating it [88] (Point 39). An alternative pathway for inhibiting GSK-3β is phosphorylation by the polo-like kinase 2 (PLK2) at Point 40 [89], which is upregulated by protocatechuic aldehyde—an Nrf2 activator shown to ameliorate symptoms in models of Parkinson’s disease [90]. We have seen fivefold upregulation of PLK2 in SKNSH glioblastoma-derived cells treated with the carnosic-acid-based Nrf2 activators PB125 and PB123 [6,55]. On the other hand, a phosphatase (PHLPP2) can deactivate p-AKT at Point 41, maintaining GSK-3β in its active form [91]. Downregulation of PHLPP2 by the phytochemical morin has been shown to prevent renal injury [91] and hepatocyte damage from acetaminophen toxicity [92]. If GSK-3β is allowed to remain in its unphosphorylated active state, it may convert FYN kinase to an active phosphorylated state, p-FYN (Point 42), whereupon p-FYN may translocate to the nucleus (Point 43) to phosphorylate Nrf2 at tyrosine-568 [93]. This marks Nrf2 for nuclear export (Point 44), for another ubiquitination, and for degradation by the proteasome (Point 45). With that, the tortuous pathway taken by the Nrf2 molecule comes to an end.

## 3. The Complexity of the Nrf2 Pathway Tells Us “One Size Does Not Fit All”

Early research in the Nrf2 field tended to be presented as a “binary” phenomenon—either Nrf2 was inducing its protective array of genes, or it was not. Phytochemicals and drugs either activated, or they did not. The degree of activation was not (and still is not) able to be quantified in terms that meaningfully translate between various studies carried out in different laboratories. Nrf2 regulates a huge repertoire of genes. Clearly, many different evolutionary pressures have driven the creation of the many control points. Management of stress is required for every tissue and organ, but the origins of the stress and the systems that may need to be protected may vary enormously from one organ to the next. Thus, it would not be surprising that triggers for Nrf2 activation may function best when under local organ control. It is highly unlikely that hundreds of genes would all need to be regulated at the same time, and to the same degree throughout the body. We now understand that redox balance is critical, so globally upregulating genes to alleviate traumatic brain injury, for example, might create redox imbalance in other organs that have not been injured. We are beginning to understand how the pathway might accommodate these situations and more.

## 4. MST1 Presents an Intriguing Alternative Route to Nrf2 Activation

The example of Wang et al. [40] discussed above and in Figure 2B illustrates the utility of “local control” versus “global control” of antioxidant enzyme expression in immune cells. Macrophages in a healthy individual may continually encounter microbial invaders in small numbers, which could become major infections. They deal with the invaders by phagocytosis and activation of an NADPH oxidase to intentionally expose the microbes to lethal oxidative stress, but the phagocyte can sustain oxidative injury in the process. It needs to strengthen its own internal protection outside the phagocytic vacuole so that it does not die along with its prey. It utilizes its own ROS sensor, Mst1—a kinase activated by the increased local production of ROS. The kinase phosphorylates Keap1, triggering the same self-ubiquitinating and self-destructive features of Keap1 that might be triggered by global oxidative stress, but doing so at the single-cell level. Wang et al. [40] speculated that the failure of this early warning system might be responsible for the decline in immunity seen with aging. Indeed, Choi et al. [94] have proposed similar phenomena in regulating the homeostasis of peripheral naïve T lymphocytes and resulting immunodeficient states in mice, while Nehme et al. [95] described several human cases of Mst1 deficiency resulting in various presentations of immune deficiency, illustrating the importance to overall health of this seemingly redundant and insignificant modification to the pathway for Nrf2 activation. These observations suggest that the mechanisms of Nrf2 activation may exist in many variations in different cell types and tissues. These parallel but alternative mechanisms might allow for some important locations to have more sensitive triggers than others, or even triggers that result in modified gene expression profiles.

## 5. Local Control May Selectively Regulate Nrf2 Effects That Are Context-Dependent

A multifunctional, far-reaching transcription factor such as Nrf2 will inevitably encounter conflicted scenarios where the best solution to a problem may not be obvious or simple. Some effects of Nrf2 activation might be helpful, while at the same time others may be harmful. That is, the most desirable gene expression profile for Nrf2’s effects may be very context-dependent. The complexity of the activation pathway may invite evolutionary solutions that could, for example, negate or even reverse regulatory effects that would in certain circumstances be counterproductive. The literature provides several examples of “good cop, bad cop” scenarios. Some of the most obvious transformations to take place in a single cell type occur in the macrophage, as these cells can be pro-inflammatory or anti-inflammatory, or participants in the cleanup of post-inflammatory damage, or in tissue remodeling. These seemingly contradictory roles of the macrophage have recently been reviewed by Jensen et al. [96] in the context of both type 1 and type 2 diabetes. With the sweeping and dramatic changes in metabolic pathways and resultant cellular functions taking place in these cells, it seems that the job of Nrf2 in regulating literally hundreds of genes that are directly or indirectly related to these changing roles would become impossible, especially if its gene expression profile were inflexible. We suggest that the pathway depicted in Figure 1 allows for the possibility of a spectrum of gene expression profiles that may be “tuned” by varying the components of the Nrf2 complex that ultimately binds to any given EpRE/ARE promoter. The affinity for the promoter and the efficiency of transcription may be modified by the assortment of post-transcriptional modifications (with combinations and permutations of multiple sites for phosphorylation, acetylation, methylation, SUMOylation, and ubiquitination) and binding partners (e.g., CBP, small MAF proteins) or binding competitors (e.g., BACH1) that the Nrf2 factor has acquired in that particular cell type. As promoter sequences have considerable variability from gene to gene, it is probable that evolution has grouped related gene families (such as cytokines) to promoter sequences that respond efficiently to Nrf2 bearing a particular set of decorations and/or partners.

## 6. Can the Activation Pathway Affect the Gene Expression Profile?

**Figure 3:**
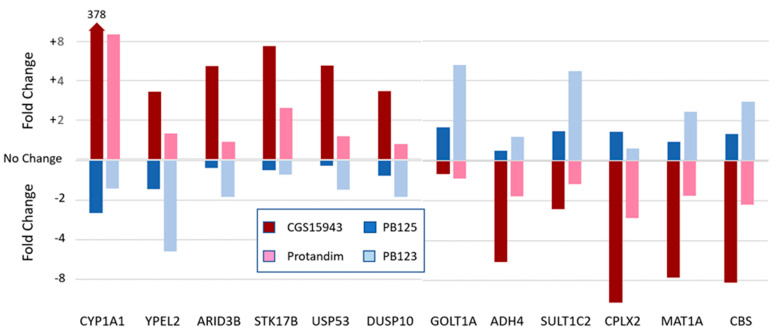
Two groups of genes are induced in opposite directions by the four Nrf2 activators, with clear similarity between the CGS15943 and Protandim pair, and clearly opposite similarity between the PB123 and PB125 pair. Details have been previously published in [97].

The competing transcriptional repressor, BACH1 (Point 34), provides another potential level of control over Nrf2’s transcriptional efficiency, as its effectiveness may also be determined by promoter sequence variants, as well as by small-molecule inhibitors or by CRM1, which can export BACH1 from the nucleus. Modifications that favor BACH1 while impeding Nrf2’s efficacy may conceivably reverse the regulatory effect from induction to repression. Figure 3 shows the response of a group of 12 genes to each of four different Nrf2 activators from experiments that we have previously described [97]. The first pair of Nrf2 activators are CGS15943 (a triazoloquinazoline adenosine receptor A2A antagonist—a pure single compound) and Protandim^®^ (a composition of five phytochemical extracts, as previously described [98]). These show induction/repression patterns that are qualitatively identical, with the first six genes being induced and the remaining six genes being repressed. The second pair of Nrf2 activators are compositions of phytochemical extracts—PB123 [6,97,99] and PB125 [97,100,101], both relying on carnosic acid as the primary activator. Here, we see the first six genes being repressed and the remaining six genes being induced. One can propose several possible explanations for this behavior. For example, another unidentified transcription factor might be activated by an unknown compound present in the less-than-pure phytochemical compositions, but the phenomenon is in fact seen most strongly with the pure, single pharmaceutical compound CGS15943. Furthermore, it would seem very unlikely that these 12 genes, in every case, would flip their response from induction to repression, or vice versa. We believe a more likely explanation may lie in the competitive involvement of the EpRE/ARE transcriptional repressor BACH1 in the regulation of these genes. Indeed, the involvement of BACH1 has been documented in the regulation of three of these genes: *CYP1A1* [102], *CBS* [103], and *MAT1A* [104]. Cystathionine beta-synthase (CBS) and methionine adenosyltransferase 1 alpha (MAT1A) are of interest because of their involvement in homocysteine metabolism and prevention of the problems that can result from hyperhomocysteinemia [105,106,107]. Decreased expression of CBS is associated with cardiovascular disease (e.g., atherosclerosis, myocardial infarction, ischemic stroke) [108] and recurrent miscarriages [109]. CBS is important not only for its role in the detoxification of homocysteine, but also for its production of hydrogen sulfide, which is itself an Nrf2 activator (Point 17) and neuroprotective agent [110,111]. MAT1A is required for the formation of S-adenosyl methionine—a methyl donor required by many biosynthetic pathways—and its deficiency is associated with liver pathologies such as alcoholic hepatitis, nonalcoholic steatohepatitis, liver cirrhosis, and cancer [112,113]. Sodium butyrate is promoted by some as a supplement for improving gut health. Recently, however, Zapletal et al. [114] have shown that through its HDAC-inhibitory activity sodium butyrate may significantly potentiate the aryl hydrocarbon-receptor-dependent expression of CYP1A1—the enzyme that converts benzo[a]pyrene to its carcinogenic metabolite [115]. Thus, all three of the genes known to have BACH1-associated regulation, and which are regulated by the CGS15943/Protandim pair in directions opposite to those seen with the PB123/PB125 pair of Nrf2 activators, could conceivably result in adverse physiological effects. Stated another way, Nrf2 activators—whether pharmaceutical-derived or phytochemical-derived—that also upregulate CYP1A1 via the aryl hydrocarbon receptor, may not be the best choices. Obviously, more work will be needed to test this hypothesis.

## 7. Network Pharmacology Versus the “One Gene, One Drug, One Disease” Paradigm

For decades the dominant paradigm for drug discovery has been to find a single compound to induce, activate, repress, inhibit, or antagonize the presumed single target responsible for causing the disease under study. Without question, this paradigm has had some phenomenal successes, but many scientists who pride themselves on being able to “think outside the box” have not managed to find their way out of this one. A serious look at Figure 1 should dispel any scientist of the idea that a single tweak of one of the 45 points identified there (and there are surely more) could “fix” the hundreds of pathological states attributed to aberrations of the Nrf2 pathway. Network pharmacology offers an alternative approach when the old paradigm fails—and its failures are rapidly becoming apparent [116]. A reason that vital functions rely on complex pathways is to maintain resilience to single-point failures at any node. Driving across a large city offers many options for getting from point A to point B, although some may be faster, more dependable, or even more expensive than others. The design and functionality of power grids is another example of the resilience and flexibility of such systems. Neither experimental knockout of Nrf2 nor transgenic supply of more copies of the gene have provided much useful information about the development of therapies for the prevention or management of cardiovascular disease, stroke, Parkinson’s or Alzheimer’s disease, infectious diseases, etc. The answers will lie in understanding and controlling the perturbations to the networks that underlie all of these diseases.

In examining Figure 1 from a network point of view, several small hubs may be seen with few (so far) known control points, centering around transcription of the *NFE2L2* gene and around translation of the Nrf2 protein. Much more activity centers around Keap1 and the sequestration and destruction of Nrf2, involving Points 9–20. Another hub centers on post-translational modification of Nrf2 and its association with binding partners (Points 21–33). Only then can Nrf2-driven gene regulation occur. Each regulated gene may also be controlled by promoter methylation and histone methylation or acetylation reactions to provide each gene with its own unique regulation. Another hub centers on shutting down Nrf2 and removing it from the nucleus for degradation (Points 34–45). Interestingly, some individual compounds or compositions have been shown to have at least some effect on Nrf2-regulated genes, even though they appear to be acting through a single hub—but that is not to say that their action is optimal, targeted, or without collateral damage (such as sulfhydryl depletion due to electrophilicity). The most comprehensive action we have seen by a single compound is by carnosic acid acting to modify Keap1 as a mild electrophile (Point 16), to activate PI3K to phosphorylate Nrf2 (Point 22), and to activate the p-AKT/p-GSK-3β/FYN pathway (Point 39), which inhibits nuclear expulsion of Nrf2, prolonging its activity in the nucleus. It is because of the ability of carnosic acid to act at three key hubs that we have based our PB123 and PB125 compositions on it.

## 8. Future Directions

The network hub that has by far attracted the most attention from researchers is the Keap1 hub. As discussed above, electrophilic modification of Keap1 effectively allows Nrf2 to enter the nucleus rather than be diverted to the proteasome. The collateral damage due to excessive or poorly targeted electrophilicity of this class of Nrf2 activators has been ignored by many but is of great concern to some [52,53,54,117,118,119,120], resulting in a search for ways to activate Nrf2 without electrophilic modification of Keap1. In addition to the aforementioned prodrug properties of carnosic acid, which greatly reduce the problem [53,55], there are other promising avenues. Liu et al. [120] studied a group of diterpenoid phytochemicals called geopyxins and found one member of the group that appears to inhibit Keap1 without electrophilic attack on C151. While it was not highly active as an Nrf2 activator, it opens the door for a new class of non-covalent phytochemical activators. Other approaches to increasing Nrf2 activity without electrophilic side effects have focused on network hubs other than Keap1. By inhibiting the FYN-dependent nuclear export (Point 43), Nrf2 can be retained longer in the nucleus, achieving a result similar to that of increasing Nrf2’s entry into the nucleus. Here, the key enzyme is GSK-3β, which phosphorylates and activates the FYN kinase to tag Nrf2 for export. GSK-3β inactivation by phosphorylation via p-AKT (Point 39) can therefore block the export. It should be noted that active GSK-3β decreases Nrf2 via an independent mechanism at Point 14, tagging it for the proteasome. Thus, active GSK-3β acts on two separate hubs to block Nrf2. GSK-3β inhibitors have become a new class of drugs aimed at multiple diseases and proposed to act through multiple pathways [121,122,123,124]. BACH1 inhibitors, discussed above at Points 34–37, offer additional ways to increase Nrf2 activation without electrophilicity concerns.

Another very promising approach has sought non-covalent activators that compete with Nrf2 for binding to Keap1. These molecules are referred to as “iKeap1” molecules, and some have shown high-affinity binding for Keap1 in ways that block its ability to bind, ubiquitinate, and degrade Nrf2. The application of artificial intelligence, machine learning, and the ability to deduce in silico three-dimensional protein structures and binding affinities (ligand-based virtual screening, or LBVS) [125] has greatly accelerated the search for inhibitors of this type of protein–protein interaction, but the need for biological confirmation of computer predictions is still essential. In 2019, Tran et al. [126] compared 21 putative iKeap1 molecules identified at the time by a variety of evaluation methods, finding that about half fell far short of expectations with regard to their ability to bind to Keap1 or to bring about Nrf2 activation. They attributed these discrepancies to inadequate reliance on biochemical and biological assays in many early studies. Boyenle et al. [127] reviewed the progress in this area of research and concluded that while the approach has great potential to screen orders of magnitude more compounds quickly and cheaply, it still has a high rate of false positives. They expect that the methodologies will improve considerably in the coming years, which gives us something to look forward to. Success stories using this approach are making their way into the literature. Kim et al. [128] used several sequential virtual screens to identify 38 candidate compounds, which were further screened by a biological Nrf2 activation assay. One compound, KKPA4026, was indeed a potent Nrf2 activator that was nontoxic below 30 µM, appeared likely to pass the blood–brain barrier, and showed good pharmacokinetics and bioavailability.

## 9. Conclusions

In the past two decades, there have been 24,000 published studies involving Nrf2, with more than 4000 of them published in 2022. Clearly, this pathway is of huge importance, and we appear to be far from a full understanding of its reaches and ramifications. We hope to see greater emphasis on subtleties that are currently lacking with regard to how subsets of the vast repertoire of Nrf2-regulated genes might be fine-tuned by other levels of sensors, and how tissues, organs, and cell types have evolved ways to provide specialized regulation depending on their unique situations. We certainly hope to see more human clinical trials to utilize and extend what has been learned in the laboratory regarding the potential therapeutic benefits that this transcription factor has to offer.
